# Dantrolene inhibits lysophosphatidylcholine-induced valve interstitial cell calcific nodule formation *via* blockade of the ryanodine receptor

**DOI:** 10.3389/fcvm.2023.1112965

**Published:** 2023-03-30

**Authors:** Christopher B. Sylvester, Farshad Amirkhosravi, Angelina S. Bortoletto, William J. West, Jennifer P. Connell, K. Jane Grande-Allen

**Affiliations:** ^1^ Department of Bioengineering, Rice University, Houston, TX, United States; ^2^ Medical Scientist Training Program, Baylor College of Medicine, Houston, TX, United States; ^3^ Department of Surgery, Houston Methodist Hospital, Houston, TX, United States; ^4^ Center for Cell and Gene, Stem Cells, and Regenerative Medicine Center, Translational and Molecular Medicine Program, Baylor College of Medicine, Houston, TX, United States; ^5^ Morsani College of Medicine, University of South Florida, Tampa, FL, United States

**Keywords:** aortic valve, calcification, lysophosphatidylcholine, dantrolene, apoptosis, ryanodine receptor, valve interstitial cell, calcific aortic valve disease

## Abstract

Calcific aortic valve disease (CAVD), a fibrocalcific thickening of the aortic valve leaflets causing obstruction of the left ventricular outflow tract, affects nearly 10 million people worldwide. For those who reach end-stage CAVD, the only treatment is highly invasive valve replacement. The development of pharmaceutical treatments that can slow or reverse the progression in those affected by CAVD would greatly advance the treatment of this disease. The principal cell type responsible for the fibrocalcific thickening of the valve leaflets in CAVD is valvular interstitial cells (VICs). The cellular processes mediating this calcification are complex, but calcium second messenger signaling, regulated in part by the ryanodine receptor (RyR), has been shown to play a role in a number of other fibrocalcific diseases. We sought to determine if the blockade of calcium signaling in VICs could ameliorate calcification in an *in vitro* model. We previously found that VICs express RyR isotype 3 and that its modulation could prevent VIC calcific nodule formation *in vitro.* We sought to expand upon these results by further investigating the effects of calcium signaling blockade on VIC gene expression and behavior using dantrolene, an FDA-approved pan-RyR inhibitor. We found that dantrolene also prevented calcific nodule formation in VICs due to cholesterol-derived lysophosphatidylcholine (LPC). This protective effect corresponded with decreases in intracellular calcium flux, apoptosis, and *ACTA2* expression but not reactive oxygen species formation caused by LPC. Interestingly, dantrolene increased the expression of the regulator genes *RUNX2* and *SOX9*, indicating complex gene regulation changes. Further investigation *via* RNA sequencing revealed that dantrolene induced several cytoprotective genes that are likely also responsible for its attenuation of LPC-induced calcification. These results suggest that RyR3 is a viable therapeutic target for the treatment of CAVD. Further studies of the effects of RyR3 inhibition on CAVD are warranted.

## 1. Introduction

Calcific aortic valve disease (CAVD), a fibrocalcific thickening of the aortic valve leaflets causing obstruction of the left ventricular outflow tract, affects nearly 10 million people worldwide, and its prevalence is increasing (1). For those who reach end-stage CAVD, the only treatment is highly invasive surgical or transcatheter valve replacement (2). The development of pharmaceutical treatments that can slow or reverse the progression of fibrosis and mineralization in those affected by CAVD would significantly advance the treatment of this disease (3).

The primary drivers of CAVD are the valvular interstitial cells (VICs), the resident fibroblasts of the valve leaflets. VICs maintain their ability to calcify *in vitro*, and thus they are often investigated as a disease model for CAVD (4, 5). Several agents have been shown to induce VIC calcification, but lysophosphatidylcholine (LPC) plays a role in VIC-mediated calcification *in vivo, in vitro*, and in humans (4, 6, 7). LPC is a lipid that makes up the main component of oxidized low-density lipoprotein. It has been implicated in a number of other inflammatory diseases, such as atherosclerosis, diabetes, and cancers (8). The mechanisms by which LPC induces VIC calcification are not fully understood but involve its conversion to lysophosphatidic acid *via* lipoprotein(a) and autotaxin (7), leading to transcriptional and phenotypical changes that drive calcific nodule formation. Further, LPC deranges ionic calcium flux in VICs *via* the ryanodine receptor (RyR), an effect that we previously showed was tied to their tendency to form calcific nodules (9). We also demonstrated that VICs primarily express RyR isotype 3 (RyR3) (9). However, the broader effects of blocking calcium flux on mitigating the activating, apoptotic, and pro-inflammatory effects of LPC are still not known.

Dantrolene, a pan-inhibitor of RyR isotypes, is an orphan drug approved for treating malignant hyperthermia, an uncommon complication of general anesthesia, and as a muscle relaxant in chronic spasticity (10, 11). Recent studies have revealed dantrolene's potential as a therapeutic for various diseases, including Alzheimer's dementia (12, 13), Duchenne's muscular dystrophy (14), and several arrhythmias (15, 16). Dantrolene works not only through the inhibition of all three ryanodine receptor isotypes (16, 17) but also *via* the inhibition of reactive oxygen species (ROS) (18). Given dantrolene's ability to prevent pathological calcium signaling and reduce ROS, we sought to determine if it would be an effective therapeutic for LPC-induced calcific nodule formation *in vitro* (9).

We previously showed that LPC mediates ionic calcium flux in VICs and that blockade of the RyR3 receptor prevents LPC-induced calcium flux (9). In this study, we hypothesized that dantrolene-mediated calcium blockade would prevent calcific nodule formation. We further analyzed the phenotypical and transcriptional effects of dantrolene on LPC-treated VICs. Overall, we conclude that dantrolene shows early promise as a pharmaceutical treatment for CAVD.

## 2. Materials and methods

### 2.1. VIC isolation and expansion

Porcine aortic VICs (paVICs) were isolated from pig hearts commercially acquired *via* a local abattoir (Animal Technologies, Tyler, TX) as previously described (9). Valves from 6 hearts were used per isolation. Briefly, aortic valves from fresh porcine hearts on ice were dissected and washed in cold phosphate buffered saline (PBS) containing 5% antibiotic-antimycotic (ABAM; Thermo Fisher Scientific 15240062) to remove blood and debris. After dissection, the valves were incubated in a solution of 500 U/ml collagenase type II (Worthington Biochemical LS004177) in DMEM (Corning 10-014-CV) with 1% ABAM for 30 min and wiped with a sterile swab to remove the endothelial cell layer. The denuded valves were minced and incubated in a mixture of 300 U/ml collagenase type III (Worthington Biochemical LS004182), 50 U/ml hyaluronidase, and 0.5 U/ml neutral protease (Stemcell Technologies 07913) in DMEM with ABAM and 25 mM HEPES (Millipore Sigma H4034) for 4 h at 37°C in an orbital shaker. The digested suspension was filtered through a 40 μm cell strainer to remove large debris, and the filtrate was centrifuged at 1500 g for 5 min to pellet the cells. The pellet was resuspended in growth medium consisting of 1:1 DMEM:F12 (Cytiva Life Sciences SH30026) with 10 mM HEPES, 1% ABAM, and 10% bovine growth serum (BGS) and plated on a growth flask. Cultures were closely monitored for contamination for the first few days after isolation. Cells were allowed up to 7 days to attach. When cells began to grow, they were passaged once and then frozen in medium consisting of 90% BGS and 10% hybridoma-grade DMSO (Sigma Aldrich D2650) until experimentation.

### 2.2. Culture conditions

Reagent preparation was done according to manufacturer directions, and culture conditions were modified from previous studies (9). Dantrolene (EMD Millipore 251680) was dissolved in hybridoma-grade DMSO in stock aliquots of 10 mM and stored at −80°C. Working aliquots were made from thawed vials and stored at −20°C to reduce freeze-thaw. LPC (Sigma-Aldrich L4129) was stored in desiccating conditions at −20°C and dissolved in absolute ethanol (EtOH) at 10 mM just prior to use. For some experiments, osteogenic medium (OM) consisting of 10 nM dexamethasone (Sigma-Aldrich D4902) and 10 mM β-glycerophosphate (Sigma-Aldrich G9422) was used as a comparison to LPC. Reagents were diluted to final concentrations in low-serum medium (1:1 DMEM:F12 with 10 mM HEPES, 1% ABAM, and 1% BGS) unless otherwise specified. During experimentation, cells were exposed to one of 4 conditions, unless otherwise specified: vehicle-only (containing a volume of DMSO and ethanol each equal to the highest volumes of dantrolene and LPC, respectively, used in that experiment), LPC (with DMSO vehicle), dantrolene (with ethanol vehicle), or LPC and dantrolene.

**Figure 1:**
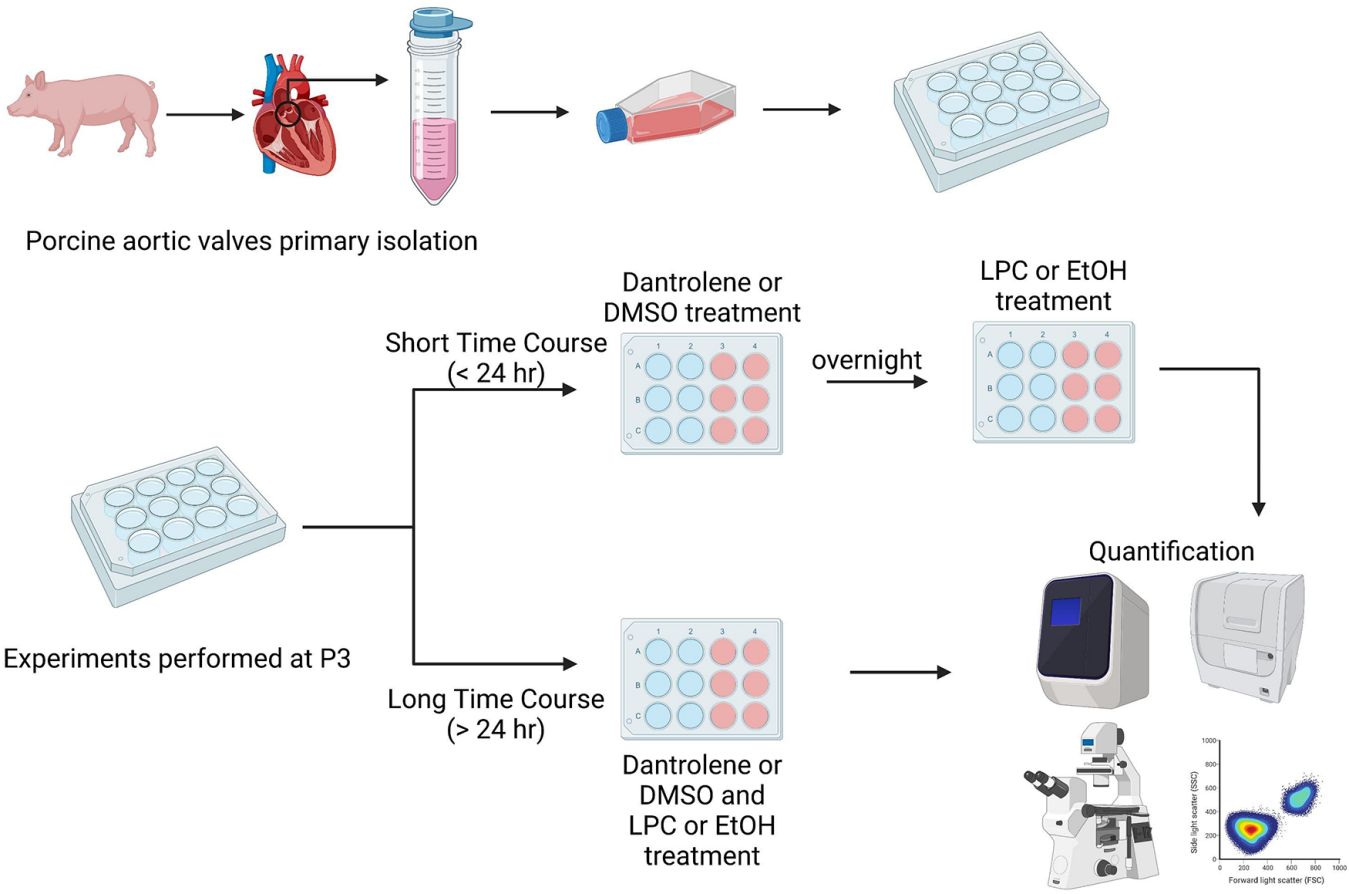
Experimental overview. Valve interstitial cells were sterilely dissected from porcine (*Sus scrofa*) aortic valves. Experiments were divided into either short or long time courses. For short time course experiments, cells were treated with dantrolene or DMSO vehicle overnight and then exposed to LPC or ethanol vehicle for less than 24 h the next day. For long time course experiments cells were treated with dantrolene or DMSO vehicle and LPC or ethanol vehicle at the same time. Media were changed every 3 days. Created with BioRender.com.

When ready for experimentation, paVICs were thawed and passaged once using growth medium. Once confluent, the paVICs were passaged and seeded onto the appropriate culture plates at the density desired for the experiment, described below. Cells were then exposed to low-serum medium for 1–2 days before experimentation. Experiments were broadly divided into short- and long-term categories, as illustrated in Figure 1. Short-term experiments examined rapid events due to LPC occurring less than 24 h after exposure. For short-term experiments, paVICs were pretreated with 60 μM dantrolene or DMSO vehicle overnight prior to LPC or EtOH vehicle exposure. For long-term experiments, examining events greater than 24 h after initial exposure to LPC, paVICs were simultaneously exposed to dantrolene or DMSO vehicle and LPC or EtOH vehicle, and conditions were maintained throughout the course of experimentation, changing medium every 3 days, as needed.

### 2.3. Cell viability

To determine the appropriate treatment concentration of dantrolene, we first used an alamarBlue (Bio-Rad BUF012) cell viability assay (19). Cells were treated with serial dilutions of dantrolene overnight in an incubator. A live control was treated with only low-serum medium, and a dead control was treated with 10% ethanol. The next day, the culture medium was removed, and the cells were incubated with a 10% alamarBlue solution in HEPES-buffered Hank's Balanced Salt Solution (HHBSS) in an incubator. After one hour, fluorescence was read at an excitation and emission of 530 and 590 nm, respectively, using a SpectraMax M2 plate reader (Molecular Devices). Signal was normalized to that of the live control. Cell viability curves were fitted to a four-parameter logistic curve, and the interpolated concentration that corresponded to 50% signal of the live control was used to calculate the cellular median lethal dose (LD50). The average LD50 of two independent experiments is reported.

Other cells, treated with a similar amount of dantrolene or DMSO, were treated with a Live/Dead staining kit (Invitrogen L3224) consisting of calcein-AM and ethidium homodimer. Cells were treated with a 1:1,000 dilution of calcein-AM and ethidium homodimer in HHBSS for 30 min in an incubator. After washing, the cells were imaged on an ECLIPSE Ti2E microscope (Nikon Instruments) using FITC for detecting living cells and Texas Red for detecting dead or compromised cells. Acquired images were analyzed with a custom CellProfiler (Broad Institute) pipeline to count the number of objects in each channel and determine percent viability as described elsewhere (20). Six replicates were used per group, and data from two independent experiments are displayed.

### 2.4. Calcium signaling

To quantify changes in calcium flux, we used a Fluo-8 calcium assay (Abcam ab112128) as previously described. Briefly, cells were confluently seeded onto a clear-bottomed, black-walled 96-well plate. The next day, they were exposed to either 60 μM dantrolene in DMSO or an equivalent amount of DMSO (0.6% v/v) only overnight. The following day, the medium was removed and replaced with assay buffer consisting of 1:1,000 Fluo-8 dye from stock in 1× Pluronic F127 Plus in HHBSS, provided by the kit. The cells were incubated at 37° for 30 min then 30 more minutes at room temperature. After dye loading, calcium flux was immediately measured in a TECAN Spark multimode plate reader at an excitation/emission of 490/525 nm. The appropriate signal gain was calibrated using carbachol (Abcam 141354), a calcium agonist. The plate reader's automated pipettes were then used to deliver LPC at a final concentration of 10 μM in HHBSS or an equivalent volume of 0.1% EtOH in HHBSS. Fluorescence was read every second for 5 s before adding reagents and for 25 s after adding reagents. Data are reported as the maximum fluorescence achieved after the addition of reagents minus the average of the signal before the addition of reagents, which was considered background. Six replicates were used per control group with 18 replicates per dantrolene-treated group.

### 2.5. Calcific nodules

The formation of paVIC calcific nodules was assayed as previously described (9). Briefly, paVICs were seeded onto a 48-well plate in low-serum medium at 50,000 cells/cm^2^ to yield a confluent monolayer. After two days, the medium was changed to conditioned media, consisting of DMSO only, 10 μM LPC with DMSO vehicle, or a 10 μM, 30 μM, or 60 μM dose of dantrolene with 10 μM LPC. Separate experiments were conducted using OM as a reference. Calcific nodules were allowed to form for 6 days, and calcific media were changed on day 3. On day six, after media removal, the cells were washed with PBS, fixed with 4% paraformaldehyde for 30 min, washed twice with deionized water, and left to dry overnight. The next day, samples were stained for 30 min with 40 mM alizarin red-S (Sigma-Aldrich A5533) in deionized water adjusted to a pH of 4.2–4.4. After staining, the samples were washed with deionized water 3 times until the wash was clear. Samples were imaged on the ECLIPSE Ti2E, and nodule size and number were quantified using ImageJ. Four replicates were included for each experimental group, and data from one representative experiment are reported.

### 2.6. Caspase activity

The activity of caspases 3 and 7 was measured as an analog for apoptosis using the Caspase-Glo 3/7 Assay Kit (Promega G8091). Cells were seeded confluently onto a 384-well plate and allowed to attach overnight. The next day, cells were treated with 10, 20, 40, or 80 μM LPC or ethanol control and either 60 μM dantrolene or DMSO vehicle overnight. The following day, the reagents were applied per the manufacturer's directions, and then the luminosity was detected on the SpectraMax M2 plate reader. Four replicates were included per control group, and 6 replicates were included per test group. Data from two independent experiments are reported.

### 2.7. Reactive oxygen species quantification

To quantify the production of ROS due to LPC, we used a CM-H_2_DCFDA general oxidative stress indicator (Thermo Fisher C6827). To measure ROS, paVICs were plated on 6-well plates and allowed to grow to 90% confluency in growth medium. When ready, the cells were treated with 60 μM dantrolene or DMSO vehicle overnight in low-serum medium. The next day treated media were removed, the cells washed with PBS, and then exposed to 10 μM LPC for 10–60 min in HHBSS or ethanol vehicle for 60 min in an incubator. Other dantrolene treated and untreated control groups were exposed to only HHBSS or 200 μM *tert*-butyl hydroperoxide (Thermo Fisher 180340050), a potent inducer of ROS, for 30 min. After exposure, cells were washed with PBS and loaded with 10 μM CM-H_2_DCFDA in HHBSS for 30 min in an incubator. After dye loading, the cells were lifted from their plates using 0.25% trypsin, collected in HHBSS without calcium or magnesium, and fluorescent intensity was examined using flow cytometry in an MA900 Cell Sorter (Sony). Median fluorescence intensity (MFI) was used to determine changes in ROS production. Flow data were analyzed as MFI using Flowing Software (version 2.5.1). Three replicates were included per experimental group, and data from two independent experiments are reported.

### 2.8. Quantitative real-time polymerase chain reaction

Quantitative real-time polymerase chain reaction (qRT-PCR) was carried out as previously described (9). Briefly, paVIC cultures were exposed to LPC, dantrolene, or their respective vehicles for 1 or 3 days. After experimentation, RNA was extracted using TRIzol reagent (Invitrogen 15596018) and purified using a Direct-zol RNA Microprep spin kit (Zymo Research R2062) per the manufacturer's directions, with a DNAse treatment. RNA was eluted into RNAse-free water. RNA quantity and quality were measured using a Nanodrop 2000 (Thermo Fisher). A value greater than 1.8 was considered acceptable for 260/280 and 260/230 ratios. Reverse transcription was conducted using a High-Capacity cDNA Kit (Applied Biosystems 4368813). qRT-PCR was performed on a CFX96 Real-Time PCR System (Bio-Rad) using probes listed in Supplementary Table S1. Three to 6 replicates were used per experiment.

### 2.9. RNA sequencing

Total RNA was extracted from paVICs using the same methods as for qRT-PCR. Three experiments, each using cells from separate isolations, were conducted on different days, and each experiment had one replicate per group. Purified RNA samples were sent to Novogene Corporation United States (Sacramento, CA), which performed quality control, library construction, sequencing, and data cleaning. RNA sequencing was conducted using Illumina platforms. After the data were received, annotation was carried out for *Sus scrofa* using *STAR* (v2.7.9), and batch effect adjustment was carried out using the ComBat function of the *SVA* package in R (21, 22). Differential gene expression was determined using DESeq2 (v1.32.0) in R (23). Data visualization was performed in R using the following: heatmaps were generated using *ComplexHeatmap* (v2.8.0), volcano plots were generated using *EnhancedVolcano* (v1.11.5), and finally GO, WikiPathways and KEGG pathway analysis was carried out with *enrichplot* (v1.12.3) (24, 25). The data discussed in this publication have been deposited in NCBI's Gene Expression Omnibus (26) and are accessible through GEO Series accession number GSE227229.

### 2.10. Statistical methods

Statistical analysis was performed using R version 4.1 (27). Quantitative data are expressed in the text as mean ± standard deviation and graphically as box-and-whiskers indicating median (crossbar), interquartile range (hinges), and 1.5 times interquartile range (whiskers). Experiments were repeated at least twice with the number of replicates specified unless otherwise noted. Statistical analysis was performed using *n*-way ANOVA with *post hoc* Tukey's test or Dunnett's test as appropriate. Significance was called at *P* = 0.05.

## 3. Results

### 3.1. Dantrolene has minimal toxicity on paVICs

**Figure 2:**
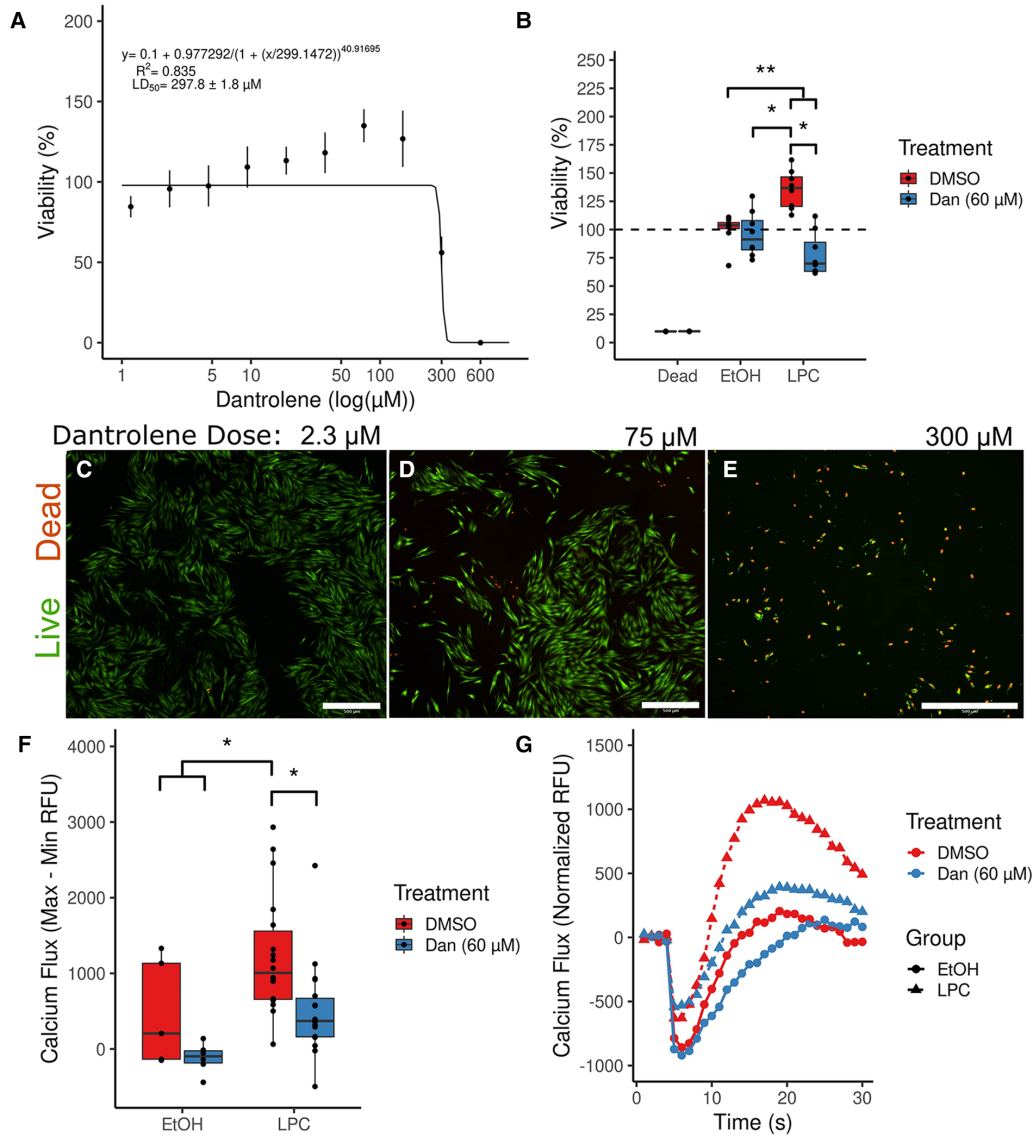
Dantrolene allows for nontoxic inhibition of calcium signaling in porcine aortic valve interstitial cells. (**A**) An alamarBlue serial dilution fitted to four parameter logistic regression calculates the cellular median lethal dose of dantrolene at 297.8 μM. (*n* = 8 per point; data are representative of two independent experiments) (**B**) Dantrolene reduces increased cellular metabolism due to LPC. (Dead group was treated with 10% EtOH; *n* = 12 per group) (**C–E**) Live/Dead staining confirms the results of the alamarBlue assay in (**A**). (**F**) Dantrolene inhibits LPC-induced calcium flux. (*n* = 6–18 per group) (**G**) Representative curves from (**F**). (*indicates *P* < 0.05 *via* Tukey's test between the indicated groups; **indicates *P* < 0.05 between all the indicated comparisons by Tukey's test; scale bar is 100 μM; Dan, dantrolene; LPC, α-lysophosphatidylcholine; RFU, relative fluorescent units; DMSO, dimethyl sulfoxide; EtOH, ethanol).

The paVICs demonstrated an LD50 of 297.8 ± 1.8 μM to dantrolene using an alamarBlue assay (Figure 2A). The cells demonstrated good viability until about 150 μM dantrolene and showed much lower viability due to high toxicity at greater doses. A dose of 600 μM regularly killed all cells in the sample. Since alamarBlue detects mitochondrial activity, treatments that stress the cells can result in a slightly elevated signal from a live (non-treated) control. Therefore, we confirmed our results using a Live/Dead stain with microscopic examination (Figures 2C–E). While there was very low baseline cell toxicity at low doses (Figure 2C), increased toxicity could be observed beginning at 75 μM (Figure 2D). At 300 μM, nearly all the cells in the sample were dead or had a compromised membrane (Figure 2E). Based on these results, we chose to use a dose of 60 μM dantrolene or less in all further experiments, which allowed for good viability with minimal interference with other assays.

We further examined the effects of our reagent combinations on paVICs using an alamarBlue assay (Figure 2B). Dantrolene (60 μM) had no effect on viability compared to vehicle-treated cells (95.9 ± 19.9% vs. 100.0 ± 13.6%, *P* = 0.992). Meanwhile, treatment with 10 μM LPC showed an apparent increase in viability compared to all other groups (135.5 ± 17.0%, *P* < 0.001, all comparisons). This apparent increase in viability could be due to increased cell proliferation or mitochondrial stress, either of which would indicate that LPC negatively affects paVIC quiescence. Treatment with dantrolene and LPC reversed this increase in alamarBlue signal (78.0 ± 19.3%, *P* < 0.001 vs. LPC). Treatment with both dantrolene and LPC decreased the viability of paVICs compared to vehicle-only treatment (*P* = 0.042) but not dantrolene only (*P* = 0.151), indicating that there may be some slight toxicity in the combination treatment.

### 3.2. Dantrolene inhibits LPC-induced calcium flux in paVICs

We investigated the effects of 60 μM dantrolene on LPC-induced calcium flux using Fluo-8, a calcium-sensitive dye. We found that overnight pretreatment with dantrolene inhibited LPC-induced calcium flux (Figures 2F–G). Treatment with LPC caused a significant increase in calcium flux compared to vehicle-treated cells (476.4 ± 551.2 vs. −103.3 ± 321.2, *P* = 0.028). Pretreatment with 60 μM dantrolene successfully blocked this increase in calcium flux due to LPC (95.0 ± 301.6, *P* = 0.011). Representative curves for each group can be seen in Figure 2G.

### 3.3. Dantrolene reduces calcific nodule formation of paVICs *in vitro*

**Figure 3:**
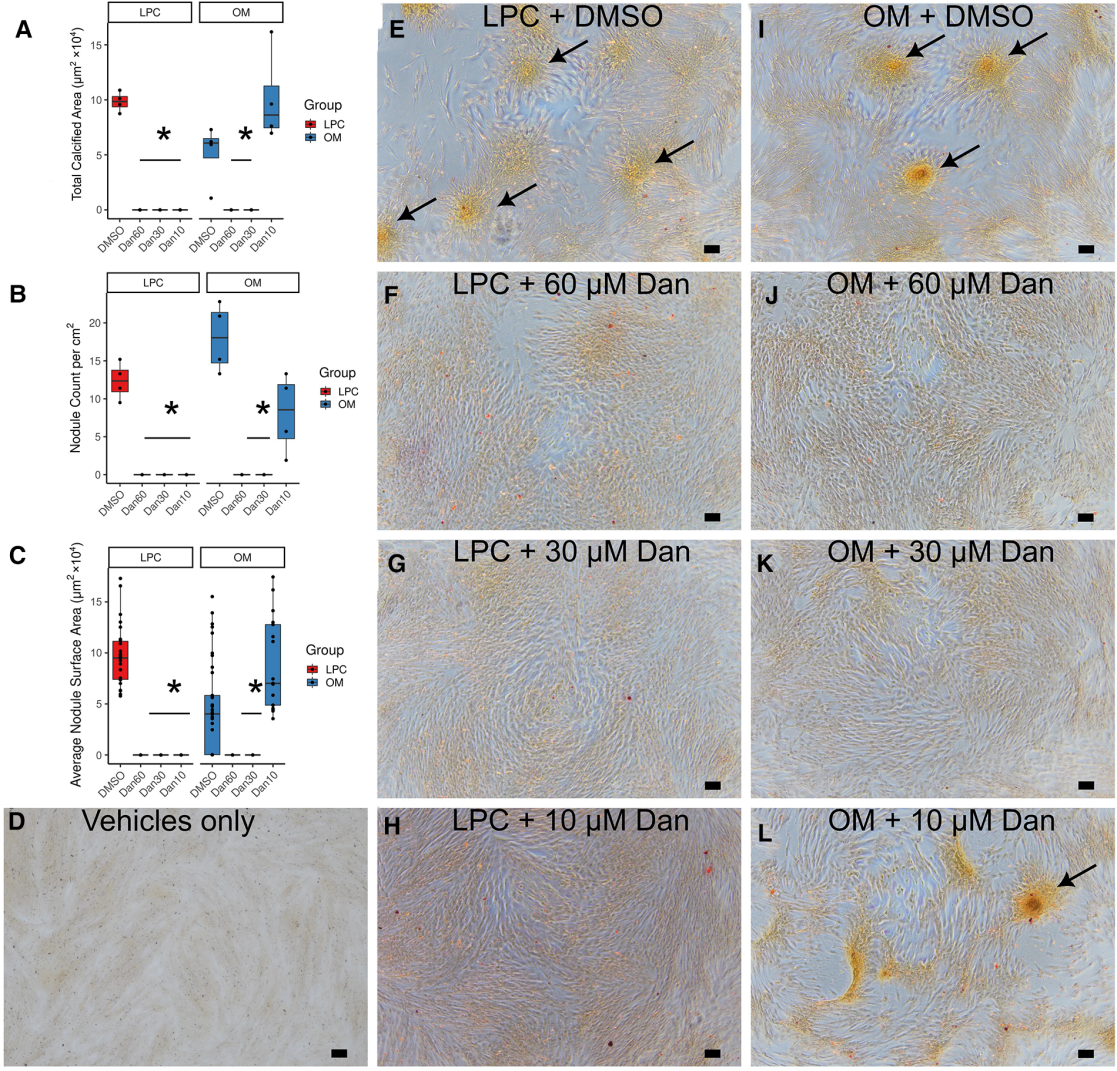
Dantrolene reduces *in vitro* calcific nodule formation by paVICs due to LPC and OM. Dantrolene reduces total calcified area (**A**), nodule number per well (**B**), and average nodule surface area (**C**). (**D**) Vehicle-only control (0.1% ethanol and 0.6% DMSO). (**E–L**) Red-yellow stain is alizarin red. (**E**) LPC in DMSO-treated paVICs. 10 μM LPC with (**F**) 60 μM, (**G**) 30 μM, and (**H**) 10 μM dantrolene. (**I**) OM in DMSO-treated paVICs. (**J**) 60 μM and (**K**) 30 μM dantrolene with OM. (**L**) Breakthrough nodules can be seen in 10 μM dantrolene with OM. (*n* = 4 per group for all experiments in this figure; (**C**) shows each nodule as a point; * indicated *P* < 0.05 from DMSO-treated control *via* Dunnett's test; arrows indicate areas of calcification; scale bar is 100 μM; paVICs, porcine aortic valve interstitial cells; LPC, α-lysophosphatidylcholine; OM, osteogenic medium).

Dantrolene inhibited calcific nodule formation of paVICs due to 10 μM LPC (Figures 3A–H). Treatment with dantrolene at doses of 60, 30, or 10 μM decreased total calcified area (Figure 3A), nodule number (Figure 3B), and nodule size (Figure 3C) compared to LPC in vehicle-treated cells. To confirm dantrolene's calcific nodule inhibiting ability, we repeated these experiments with osteogenic medium (OM) (Figures 3A–D,I–L). Comparable results were found with OM, but a 10 μM dose of dantrolene was ineffective at preventing OM-induced nodules (Figure 3L).

### 3.4. Dantrolene reduces LPC-mediated apoptosis

**Figure 4:**
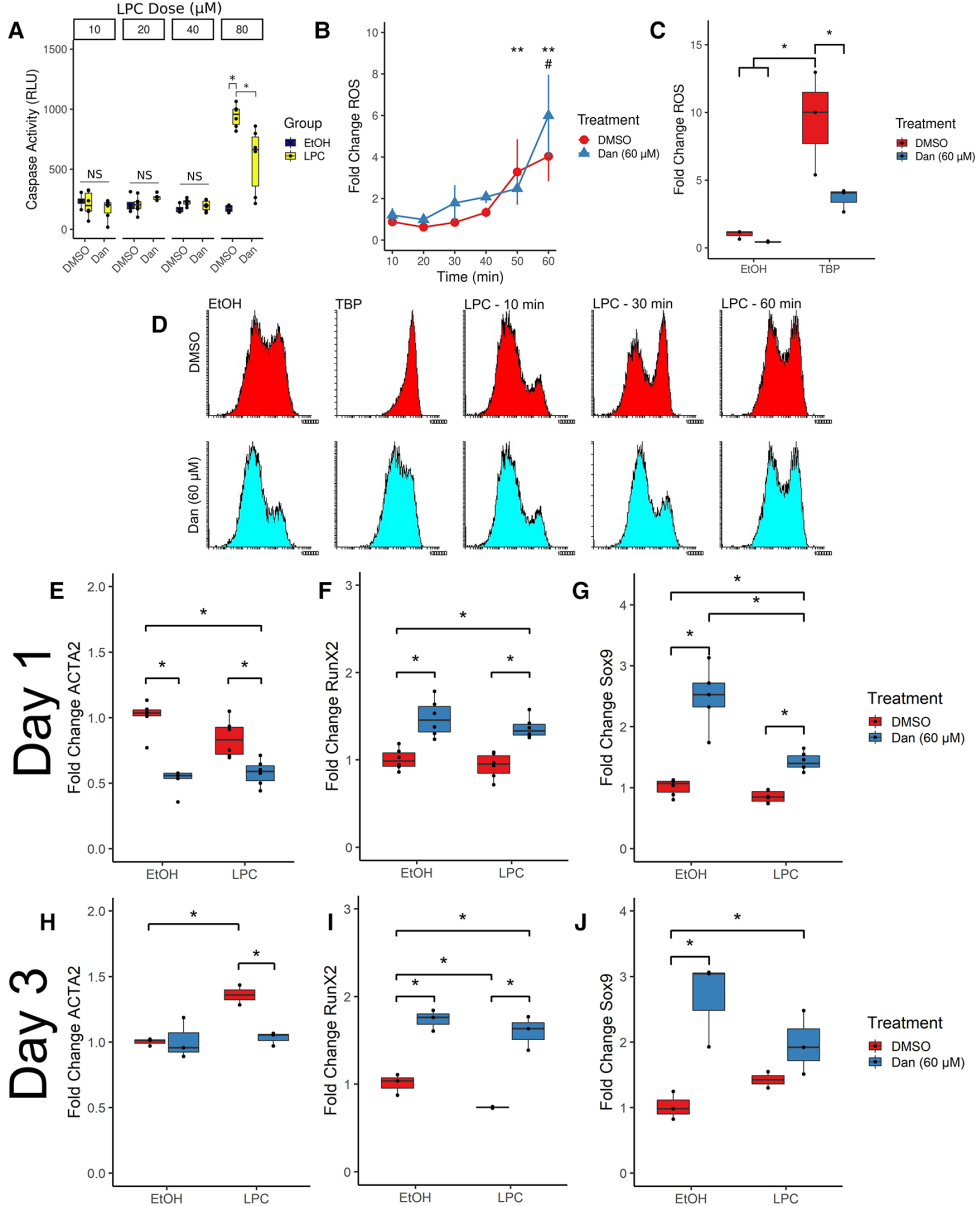
Dantrolene induces a number of phenotypical changes compared to cells treated with LPC. (**A**) Dantrolene inhibits LPC-mediated apoptosis. (*n* = 8–12 per group) (**B**) Dantrolene has no effect on LPC-induced ROS (*n* = 6 per group; **indicates significances from untreated control in dantrolene-treated [blue] group; # indicates significance reached DMSO-treated [red] group) but (**C**) inhibits ROS due to *tert*-butyl hydroperoxide. (*n* = 3 per group) (**D**) representative fluorescent intensity histograms from (**B**). (**E,H**) Dantrolene inhibits *ACTA2* expression at 1 and 3 days compared to LPC. (**F,I**) Dantrolene upregulates *RUNX2* expression in paVICs. (**G,J**) Dantrolene upregulates *SOX9* expression in paVICs. (*n* = 6 for day 1 timepoints and *n* = 3 for day 3 timepoints; *indicates *P* < 0.05 *via* Tukey's test between the indicated groups; paVIC, porcine aortic valve interstitial cell; LPC, α-lysophosphatidylcholine; RLU, relative luminescence units; ROS, reactive oxygen species; Dan, dantrolene; DMSO, dimethyl sulfoxide; EtOH, ethanol; TBP, tert-butyl hydroperoxide).

LPC induces apoptosis in various cell types, and apoptotic cells may contribute to calcific nodule formation. We measured apoptosis in paVICs *via* caspase 3/7 activity (Figure 4A). Since 10 μM of LPC did not induce measurable levels of apoptosis in paVICs, we performed serial doublings and determined that 80 μM of LPC was sufficient to induce apoptosis without signal interference from the vehicle. At 80 μM, LPC significantly induced caspase 3/7 activity compared to the vehicle-only control as measured by a luminescent assay (942.8 ± 94.4 vs. 172.3 ± 31.6 RLU, *P* < 0.001). In cells pretreated with dantrolene, significantly less caspase activity was measured (577.4 ± 273.0 RLU, *P* = 0.017), but cells treated with 80 μM LPC + 60 μM dantrolene treatment still had higher caspase activity than the vehicle-only control (*P* = 0.018). These results indicate that dantrolene partially inhibits the apoptotic effects of LPC on paVICs.

### 3.5. Dantrolene has no effect on LPC-induced ROS

LPC induces ROS in a number of cell types, and dantrolene has been shown to inhibit ROS formation. Therefore, we hypothesized that dantrolene would inhibit LPC-mediated ROS. We measured ROS formation *via* flow cytometry using a ROS-sensitive fluorescent dye (Figure 4B). Compared to an untreated reference, LPC-treated paVICs displayed a significant increase in ROS at 50 min of exposure (3.3 ± 1.6-fold change from paVICs treated with medium-only, *P* = 0.014); compared to dantrolene + LPC-treated cells, which showed significantly increased ROS at 60 min (6.0 ± 2.0-fold change, *P* < 0.001). No difference was found between LPC- or dantrolene + LPC-treated groups.

Because of this unexpected result, we next confirmed the antioxidant properties of dantrolene using another ROS-inducing agent. paVICs were treated with 60 μM dantrolene or DMSO vehicle and 300 μM *tert*-butyl hydroperoxide (TBP), a potent inducer of ROS (Figure 4C). TBP induced a strong ROS response compared to the vehicle-only control (9.46 ± 3.8-fold change vs. 1.0 ± 0.3, *P* = 0.003). Treatment with dantrolene inhibited this ROS production (3.6 ± 0.9-fold change, *P* = 0.028). ROS production from treatment with dantrolene was not different from the vehicle-only group (0.45 ± 0.05, *P* = 0.99). Representative histograms for the ROS flow cytometry can be seen in Figure 4D. These results indicate that dantrolene does not inhibit LPC-mediated ROS but does inhibit ROS induced by other sources.

### 3.6. Dantrolene inhibits *ACTA2* expression

Activation of paVICs as measured *via* αSMA (gene symbol: *ACTA2*) has long been considered a disease hallmark and a pro-CAVD phenotype. Tissue culture polystyrene artifactually induces this expression of αSMA, and factors that reduce its expression are believed to be beneficial in CAVD. We measured *ACTA2* expression at 1 and 3 days of exposure to LPC ± dantrolene *via* qRT-PCR. At 1 day, co-treatment with LPC and dantrolene significantly reduced *ACTA2* expression relative to both 10 μM LPC alone (0.8 ± 0.1 vs. 0.6 ± 0.1 fold change, *P* = 0.006) and vehicle control (1.0 ± 0.1 fold change, *P* < 0.001) (Figure 4E). Even in the absence of LPC, treatment with 60 μM dantrolene significantly reduced *ACTA2* expression compared to vehicle control (0.5 ± 0.1-fold change, *P* < 0.001).

By 3 days, *ACTA2* expression was consistent between the vehicle-only and 60 μM dantrolene groups (1.0 ± 0.0 vs. 1.02 ± 0.2 fold change, *P* > 0.99). The 10 μM LPC group had significantly higher *ACTA2* expression (1.35 ± 0.1 fold change, *P* = 0.031) than the vehicle control, the 60 μM dantrolene group (*P* = 0.032), and the dantrolene + LPC group (1.03 ± 0.1 fold change, *P* = 0.048) (Figure 4H). These results demonstrate that 60 μM dantrolene effectively reduces *ACTA2* expression in paVICs.

### 3.7. Dantrolene upregulates osteoblastic regulatory genes

Osteoblastic differentiation of paVICs drives valve calcification, so we examined the expression of the key regulatory factors Runt-Related Transcription Factor 2 (gene symbol: *RUNX2*) and *SRY*-Box Transcription Factor 9 (*SOX9*) using qRT-PCR at 1 day (Figures 4F–G) and 3 days (Figures 4I–J). We found that both *RUNX2* and *SOX9* are significantly upregulated at both time points due to 60 μM dantrolene.

*RUNX2* expression was significantly upregulated with 60 μM dantrolene treatment compared to vehicle control at 1 day (1.5 ± 0.2 vs. 1.0 ± 0.1-fold change, *P* < 0.001) and 3 days (1.74 ± 0.1 vs. 1.0 ± 0.1, *P* < 0.001). Treatment with 10 μM LPC did not significantly affect *RUNX2* expression at 1 day (0.9 ± 0.1, *P* = 0.702) but resulted in decreased *RUNX2* expression by 3 days (0.73 ± 0.0, *P* = 0.047). Co-treatment with dantrolene and LPC significantly increased *RUNX2* expression over LPC treatment alone at 1 day (1.4 ± 0.1, *P* < 0.001) and 3 days (1.6 ± 0.2, *P* < 0.001).

Regarding *SOX9*, dantrolene similarly increased its expression compared to vehicle control at both 1 day (2.5 ± 0.5, *P* < 0.001) and 3 days (2.7 ± 0.7, *P* = 0.006). LPC did not affect *SOX9* expression compared to vehicle control at 1 or 3 days. Co-treatment with dantrolene + LPC increased *SOX9* expression compared to LPC alone at 1 day (1.4 ± 0.1 vs. 0.85 ± 0.1-fold change, *P* < 0.001), but this effect was not significant at 3 days. Overall, these data show that dantrolene increases the expression of the gene regulation factors *RUNX2* and *SOX9*.

### 3.8. Characterization of differentially expressed genes in RNAseq analysis

**Figure 5:**
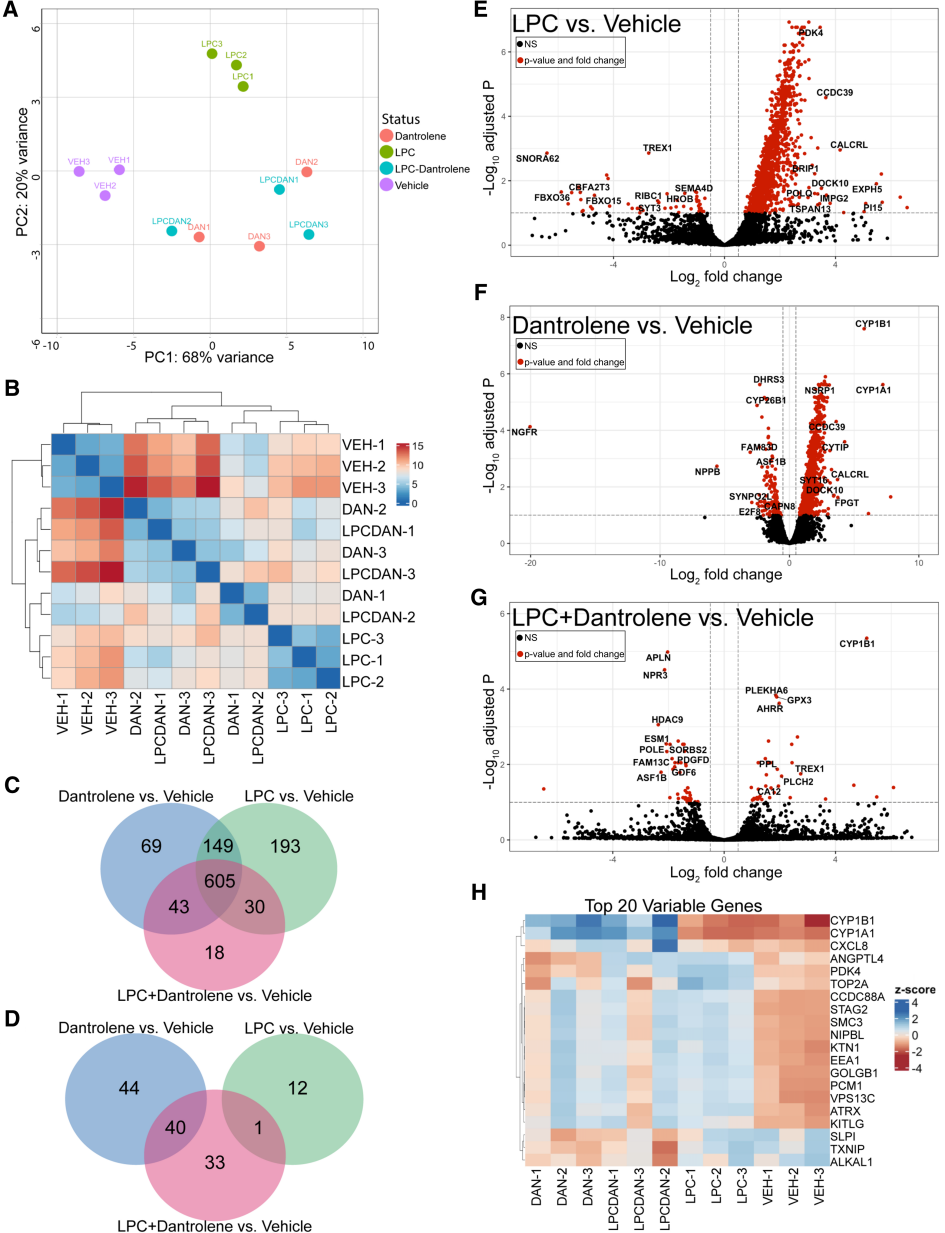
Dantrolene induces cytoprotective changes in paVICs compared to LPC. (**A**) Principal component analysis shows grouping of the vehicle, LPC controls, and dantrolene-treated replicates. (**B**) Sample distance heat map. (**C**) Dantrolene and LPC have commonly and uniquely upregulated genes. (**D**) Dantrolene and LPC alone do not share any downregulated genes. (**E–G**) Volcano plots of differentially regulated genes between LPC, dantrolene, and combination treatments. (**H**) Heat map of the top 20 differentially regulated genes. (*n* = 3 per condition; paVIC, porcine aortic valve interstitial cell; LPC, α-lysophosphatidylcholine).

Given the broad signaling and regulatory implications of calcium blockade and upregulation of *RUNX2* and *SOX9*, we used RNAseq to survey the global regulatory changes caused by treatment with LPC and dantrolene. Principal component analysis of the *ComBat*-adjusted treatment groups (Figure 5A) showed close clustering of vehicle-only treated samples, separate from those treated with 10 μM LPC, which also clustered together. Meanwhile, those treated with dantrolene or LPC + dantrolene clustered together, suggesting that these samples shared more similar gene expression profiles to one another than to the LPC-treated group. The sample distance heat map (Figure 5B) affirmed this relationship between samples.

Venn diagrams of differentially expressed genes with *P*_adj_ < 0.05 demonstrated that 690–980 genes were upregulated, and 10–85 genes were downregulated in all treatment groups compared to vehicle controls. The majority (approximately 62%–87%) of the significantly upregulated genes were shared between the three treatment groups (Figure 5C), but each group also had uniquely upregulated genes. In contrast, a Venn diagram of downregulated genes (Figure 5D) showed that the LPC group had no commonly downregulated genes compared to the dantrolene group. Further, the LPC + dantrolene group had only one downregulated gene in common with the LPC group, suggesting that dantrolene-mediated downregulation of genes dominated gene downregulation. All significantly different genes can be found in Supplementary Data Sets A–F.

Volcano plots of genes with a *P*_adj_ < 0.05 (Figures 5E,F) showed that LPC strongly promoted gene upregulation, as did dantrolene, to a lesser extent. The volcano plot of differentially regulated genes between the LPC + dantrolene group and the LPC group (Figure 5G) revealed many interesting genes that may provide insight into the regulation of calcification in valve disease. Notably upregulated genes include *CYP1B1*, a cytochrome P450 family enzyme shown to be atheroprotective (28, 29) and associated with decreased oxidative stress and inflammation (30); *BCL3*, an NFκB inhibitor associated with atherosclerotic changes (31); *IL6R*, the interleukin-6 receptor, mutations of which have been implicated in increased vulnerability to CAVD (32, 33); and *NQO1*, an NAD(P)H oxidoreductase for which upregulation has been linked with decreased calcification (34). Notably downregulated genes of interest include a DNA histone deacetylase, *HDAC9*, which has been associated with vascular smooth muscle cell calcification (35, 36); the angiotensin II receptor, *AGTR1*, which in addition to its role in blood pressure and general cardiovascular disease has also been associated with aortic stenosis and calcification (37); an actin organization gene, *SORBS2*, which has been implicated in exosome regulation (38) and myxomatous mitral valve disease (39); and the collagen synthesis enzyme prolyl-4-hydroxylase, *P4HA1*, which is induced by hypoxia inducible factor 1 (HIF1) (40), a known contributor to CAVD (41).

Analysis of the top 20 differentially regulated genes between all groups (Figure 5H) yields other interesting gene changes. The top 2 differentially regulated genes, *CYP1B1* and *CYP1A1*, cytochrome p450-family enzymes have been shown to be atheroprotective in endothelial cells (28, 29), were upregulated in all of the dantrolene-treated groups, regardless of treatment with LPC, but not in any of the other groups. The inflammatory regulator *CXCL8*, which encodes for interleukin-8, was comparatively downregulated in the vehicle control and LPC-treated groups. *ANGPTL4*, a neovascularization gene with a role in lipid metabolism (42, 43), was upregulated in both LPC treated groups and downregulated in the dantrolene only and vehicle control groups. Other genes that were strongly differential were regulators of DNA replication and repair (*TOP2A*, *STAG2*, *SMC3*, *NIPBL*) and organelle movement (*KTN, EEA1*, *PCM1*, *VPS13C*), inflammation (*SLPI*), and oxidative stress (*TXNIP*). Overall, these results suggest that dantrolene upregulates genes protective of calcification in paVICs while reducing the expression of inflammatory and oxidative genes.

### 3.9. KEGG gene enrichment analysis

**Figure 6:**
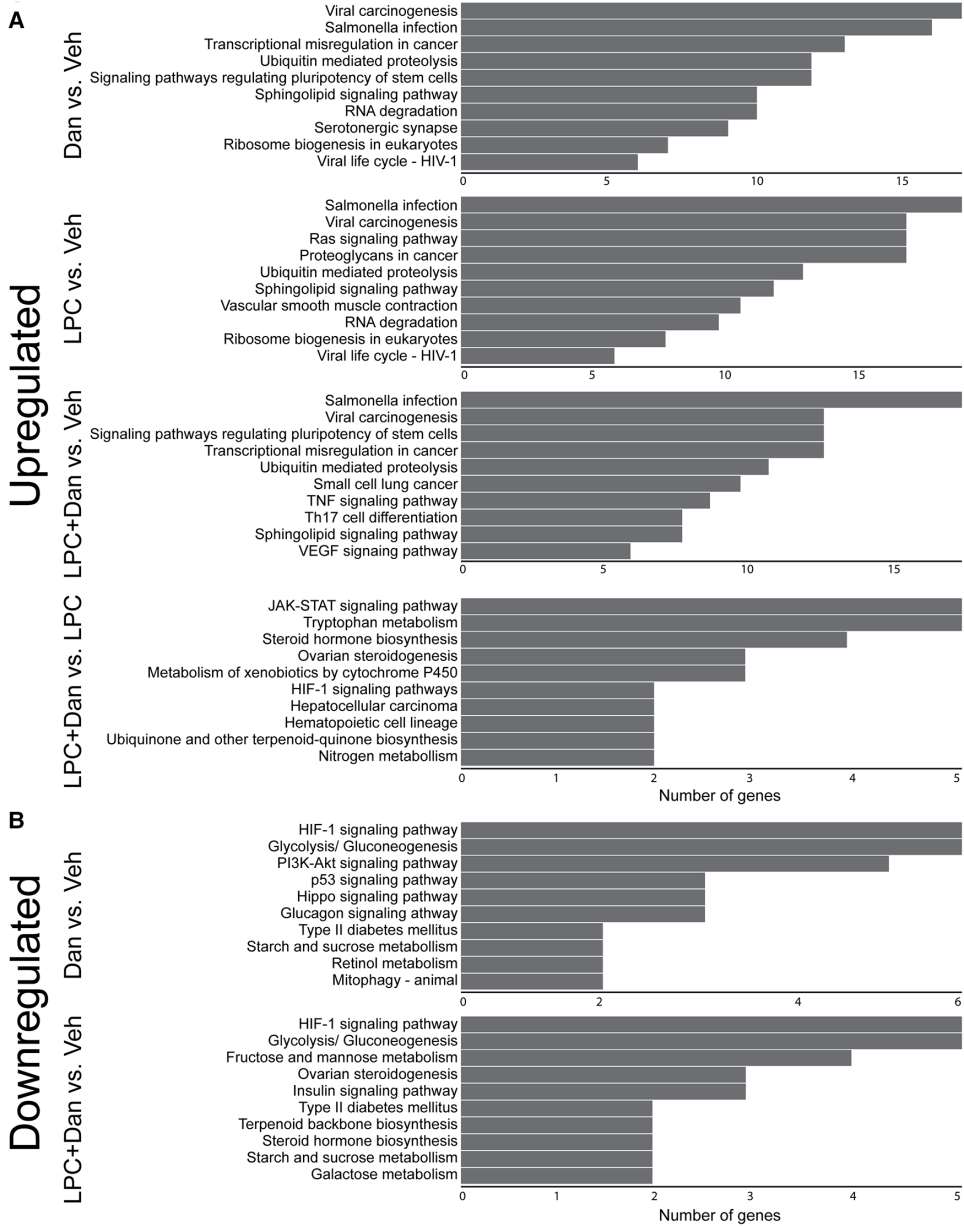
KEGG enrichment analysis of (**A**) upregulated and (**B**) downregulated genes. (*x*-axis shows number of genes differentially regulated; LPC, α-lysophosphatidylcholine, Dan, dantrolene).

KEGG pathway analysis revealed that the most upregulated genes in all groups were related to viral carcinogenesis and salmonella infection (Figure 6A). These pathways typically involve inflammation, apoptosis regulation, and cell proliferation, which overlap with the phenotypes demonstrated here. Ubiquitin-mediated proteolysis and a number of other signaling pathways appeared in all groups. Uniquely, the LPC-only group had upregulated genes relating to smooth muscle cell contraction, and the LPC + dantrolene group showed upregulation of T_H17_ cell signaling and VEGF signaling. When comparing the LPC + dantrolene group to the LPC-only group, few genes were differentially regulated with only 1–3 genes per pathway. Notably enriched pathways involved Jack-STAT signaling, cytochrome p450, and HIF.

Regarding downregulated genes (Figure 6B), the LPC-only group did not show clustering of gene pathways. However, the dantrolene-only group showed HIF1-pathway genes as the most downregulated, a change that persisted in the LPC + dantrolene group. Both dantrolene-treated groups showed a downregulation of genes relating to glycolysis/gluconeogenesis and other sugar metabolism pathways. The dantrolene-only group showed a downregulation of genes pertaining to vascular smooth muscle cell contraction, contrasting with its upregulation in the LPC-only group. The LPC + dantrolene group compared to the LPC-only group again had few pathways that were less enriched (data not shown), but notably genes in pathways relating to HIF, TGF-β, actin contraction, renin-angiotensin signaling, and osteoclast differentiation were downregulated. Overall, these results suggest multifaceted changes in paVICs due to LPC and dantrolene and highlight dantrolene's complex regulation of the HIF1 pathway.

## 4. Discussion

**Figure 7:**
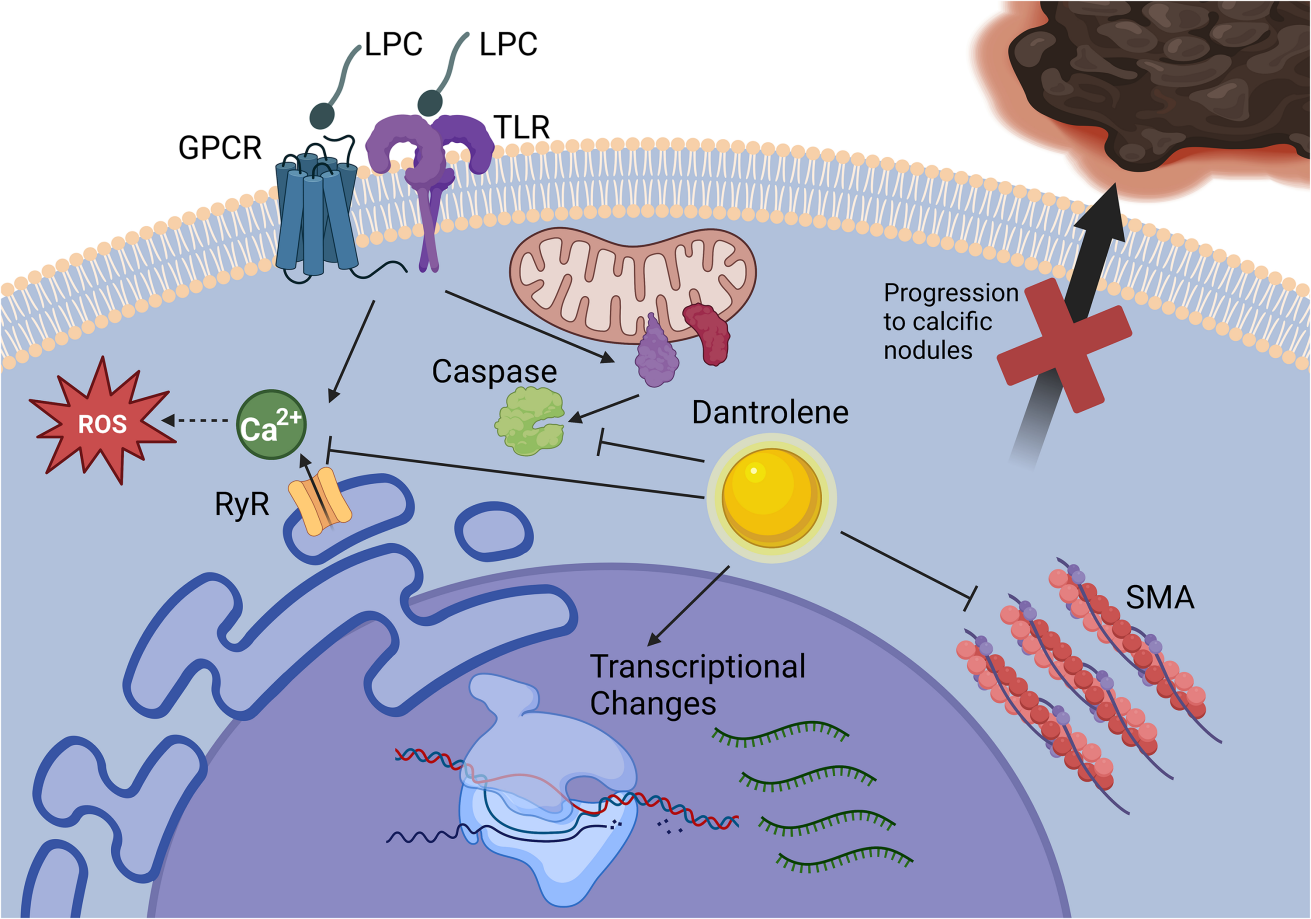
Dantrolene prevents paVIC calcific nodule formation due to LPC. Dantrolene inhibits RyR3-mediated calcium flux due to LPC and reduces LPC-associated apoptosis, SMA transcription, and progressions of paVICs to calcific nodules *in vitro*. Of note, it does not prevent LPC-associated ROS. Further, dantrolene causes global gene transcription changes relating to inflammation, cell motility, and cell metabolism. Created with BioRender.com. (paVIC, porcine aortic valve interstitial cell; LPC, α-lysophosphatidylcholine; RyR, ryanodine receptor; SMA, smooth muscle actin).

While CAVD is a common cause of morbidity and mortality, much about the mechanisms of valve calcification remains unknown. We previously showed that the phospholipid LPC induced the formation of calcified nodules by VICs *in vitro* in a calcium-dependent manner *via* the ryanodine receptor (9). However, the mechanisms by which LPC and other lipids induce calcification in VICs and the effects of calcium blockade remain unknown. Elucidation of these mechanisms and identifying a pharmacological agent that can stop or slow the progression of CAVD would benefit hundreds of thousands of people progressing toward the need for invasive aortic valve replacement. Building upon our prior work showing that the ryanodine receptor was a viable target for attenuating VIC calcification, we investigated dantrolene, an FDA-approved inhibitor of the ryanodine receptor, as a pharmacological inhibitor of LPC-induced VIC calcification (Figure 7).

First, we demonstrated that dantrolene prevents LPC-induced calcium flux in paVICs and that this calcium blockade at RyR3 was associated with decreased nodule formation. We found that calcium blockade and calcific nodule inhibition occurred without excess cell death. We also found that dantrolene inhibited calcification due to osteogenic medium, which ryanodine-mediated calcium blockade alone was unable to do (9). These results further support the hypothesis that dantrolene blockade of calcium flux *via* the ryanodine receptor prevents *in vitro* calcification but that it may also prevent calcific nodule formation *via* other mechanisms. To build upon this proof-of-concept, we further examined the effects of dantrolene on several pro-calcific pathways.

We examined VIC apoptosis, a major characteristic of valve calcification (44), and found that apoptosis only occurred in VICs at higher doses of LPC (80 μM) and that dantrolene reduces apoptotic activity due to LPC as measured by caspase 3/7 activity. Loss of calcium ion regulation by the endoplasmic reticulum is a potent driver of apoptosis (45), and others have shown that preventing LPC-mediated calcium flux prevents cell apoptosis (46). These results give further credence to the hypothesis that calcium blockade *via* dantrolene is a viable mechanism of attenuating CAVD and CAVD-related apoptosis. To pursue this mechanism further, we studied the effect of dantrolene on LPC-induced ROS production.

Despite having known antioxidant properties, in our study dantrolene did not prevent the induction of ROS by LPC, which has been shown by others to be caused by calcium signaling action on NADPH oxidase 5 (NOX5) (47, 48). However, phosphorylation of NOX5 can sensitize it to lower doses of calcium (49). Since the calcium blockade from dantrolene did not appear to be complete when LPC was applied, it is possible that this sensitization effect contributed to the persistent ROS production. Interestingly, dantrolene did reduce ROS production due to TBP, a potent inducer of ROS. These findings suggest that dantrolene can inhibit LPC-induced ROS from calcium release but not other sources, such as the mitochondria (50). Next, we assessed the link with paVIC activation, by examining the effect of dantrolene and LPC on *ACTA2* expression, a marker for VIC myofibroblast phenotype *via* αSMA.

We found that dantrolene reduced *ACTA2* expression by paVICs compared to LPC treatment alone at both 1 and 3 days. Culturing VICs on tissue culture plastic alone is enough to cause them to express αSMA, meaning that additional treatments must overcome an already strong background of expression. We found that LPC did not significantly induce *ACTA2* expression until at least 3 days of culture. To further investigate the role of cytoskeletal changes due to LPC and dantrolene, we examined filamentous actin morphology to determine if other aspects of the cytoskeleton were affected by treatment. However, we found no difference between groups (Supplementary Figure S1). We also examined the effects of dantrolene and LPC in 3-dimensional culture and found that LPC significantly upregulated *ACTA2* expression compared to control but that dantrolene did not completely reverse this effect, indicating that more complex interactions may take place in the native cellular environment (Supplementary Figure S3D). Increased expression of αSMA in VICs is indicative of an activated, contractile phenotype that contributes to CAVD progression (51, 52). The reversal of its expression by dantrolene suggests that dantrolene reverses the overall pro-calcific phenotype in VICs, which likely contributes to its anti-calcific effects. To follow up on the idea of broad phenotypical changes due to LPC and dantrolene, we examined the expression of *RUNX2* and *SOX9*, which regulate a number of genes that govern calcification and chondrogenesis.

We found that both *RUNX2* and *SOX9* were upregulated by dantrolene at 1 and 3 days. While we initially examined *RUNX2* and *SOX9* as pro-calcific factors, a literature review shows that their biology is more complex than can be generalized. While *RUNX2* does seem to promote VIC calcification, *SOX9* inhibits *RUNX2* and the nuclear localization of *SOX9* protects VICs from calcification (53). Recent studies have shown that RUNX2 localizes to calcific nodules while SOX9 can be found at the edge of nodules, further highlighting the complex relationship of these genes (54). In 3-dimensional culture, we found that LPC induced upregulation of *RUNX2* but not *SOX9*, indicating that more complex interactions may take place in the native cellular environment (Supplementary Figure S3E,F). The combined decrease in expression of *ACTA2* and increased expression of the gene regulators *RUNX2* and *SOX9* suggested an overarching change in VIC phenotype due to treatment with LPC and dantrolene. Therefore, we next employed RNAseq to gain a better perspective of the global gene expression changes within calcifying VICs.

RNAseq revealed several differential effects relating to inflammation, cell motility, and cell metabolism are altered by LPC and dantrolene. Most interesting to the protective effects of dantrolene, cytochrome p450 family member genes *CYP1A1* and *CYP1B1* were upregulated in dantrolene-treated samples. Notably, *CYP1A1* and *CYP1B1* have been shown to reduce atherosclerosis (28) and vascular dysfunction (30), respectively. Dantrolene is known to be metabolized by cytochrome P450 (55). Therefore, part of dantrolene's protective effect may be brought about by genes it induces during its metabolism. This possibility further supports the idea of a multifactorial mechanism by which dantrolene may prevent calcific nodule formation in VICs.

Other interestingly upregulated genes include *BCL3*, an anti-inflammatory NFKB inhibitor (31); *KIF12*, a microtubule motor associated with reduced lip toxicity (56); the interleukin-6 receptor, mutations of which have been associated with CAVD;(32, 33) and *NQO1*, an oxidoreductase associated with decreased calcification (34). Taken together, these differentially regulated genes suggest that dantrolene creates an anti-inflammatory pro-survival environment that may contribute to the reduced apoptosis and calcification demonstrated by dantrolene-treated VICs.

Contrary to this anti-inflammatory effect produced by dantrolene, *CXCL8* [whose gene product is interleukin-8 (IL-8)] was differentially upregulated in the groups exposed to dantrolene. IL-8 has been shown to induce calcification in human VICs (57). IL-8 primarily causes neutrophil chemotaxis, a key step in inflammation. This pro-inflammatory signaling is an interesting feature in light of the other apparently anti-inflammatory effects of dantrolene in VICs. However, the role of interleukins are not confined to the immune system, and IL-8 has been linked to endothelial cell survival, angiogenesis, and extracellular matrix remodelling (58). Further, other anti-atherosclerotic calcium-modulating drugs have been shown to upregulate *CXCL8* in a non-inflammatory manner (59). Since endothelial cells and VICs are closely situated in the valves, this signaling may represent an attempt at quiescent pro-endothelial cross-talk, rather than a pro-inflammatory signal. More complex models are needed to interpret the meaning of these gene changes, however.

Several notable genes are differentially downregulated between the LPC + dantrolene and LPC-only groups. First, *HDAC9*, a histone deacetylase gene that regulates *RUNX2* and plays a role in vascular smooth muscle cells calcification (35, 60), is downregulated in the LPC + dantrolene group. The angiotensin receptor (*AGTR1*), which has been associated with aortic valve sclerosis and calcification (37), and a collagen crosslinking gene (*P4HA1*) regulated by HIF1 were also relatively downregulated by dantrolene treatment. The association to HIF1 is interesting since HIF1 pathway genes are differentially regulated on KEGG analysis of multiple treatment groups. HIF1 critically regulates disease processes in CAVD (41, 61, 62), and the downregulation of its pathway provides another potential avenue of exploration for the effectiveness of dantrolene against calcific nodule formation. Other signaling pathways that have been linked with valve calcification, such as TGF-β were also less enriched following treatment with dantrolene (63). These results offer many interesting candidates that may explain dantrolene's impact on calcification. Given the broad effects of calcium signaling, these genes will be useful candidates for further explaining the effects of dantrolene on VICs.

This study is limited in that it only uses *in vitro* techniques to examine the physiology of LPC and dantrolene on VIC-mediated calcification. *In vivo*, VICs exist within a native extracellular matrix and are surrounded by valve endothelial cells, which further critically regulate CAVD. Given the role of inflammation in CAVD progression and the number of pro-inflammatory genes implicated by our analysis, the effect of dantrolene on infiltrating leukocytes is likely also significant. Future studies should use animal models to investigate CAVD in a more complete model and establish dosing regimens for dantrolene.

In terms of translation of dantrolene as a treatment for CAVD, it has several advantages and drawbacks. Most notably, as already mentioned, it is FDA approved, meaning that it could likely be applied faster than most experimental RyR inhibitors. It is available in multiple formations, including a pill, which could easily be taken daily with other medications used to optimize the cardiovascular health of patients with CAVD. However, dantrolene has a black-box warning for liver failure (11), limiting applicability in patients with liver disease. Regular monitoring of liver enzymes is required for the duration of its treatment (11). Since it is a muscle relaxant, it can have further size effects that may hinder activities of daily living. Additionally, while dantrolene is an effective inhibitor of the RyR, it is not specific for a specific RyR isotype. We previously showed that paVICs predominantly express RyR3 (9). Thus, identification and testing of isotype 3-specific inhibitors may provide an avenue to prevent off-target effects of dantrolene, especially muscle weakness and liver toxicity. As with any drug, actual application of dantrolene clinically would require a conversation between physicians and patients about the risks of, benefits of, and alternatives to treatment.

## 5. Conclusions

In conclusion, we demonstrate the efficacy of dantrolene in attenuating LPC-mediated VIC calcification *in vitro*. Dantrolene mediates this calcification by blockade of the LPC-induced calcium flux from the ryanodine receptor, causing reduced VIC apoptosis and myofibroblast activation due to various anti-calcific gene changes. Further investigation into the efficacy of dantrolene in animal models is warranted before the clinical application of dantrolene for CAVD.

## Data Availability

The datasets presented in this study can be found in online repositories. The names of the repository/repositories and accession number(s) can be found in the article/**Supplementary Material**. The data discussed in this publication have been deposited in NCBI's Gene Expression Omnibus and are accessible through GEO Series accession number GSE227229.
