# Supplementary material for: Dantrolene inhibits lysophosphatidylcholine-induced valve interstitial cell calcific nodule formation via blockade of the ryanodine receptor
Source: Front Cardiovasc Med. 2023 Mar 30;10:1112965. doi: 10.3389/fcvm.2023.1112965 (PMC10100588; doi:10.3389/fcvm.2023.1112965)
Supplement: Supplementary file 7 [file Datasheet7.docx]

**Supplement to: Dantrolene inhibits lysophosphatidylcholine-induced valve interstitial cell calcific nodule formation via blockade of the ryanodine receptor**

Christopher B. Sylvester^1,2^, Farshad Amirkhosravi^1,3^, Angelina S. Bortoletto^2,4^,

William J. West III^1,5^, Jennifer P. Connell^1^, K. Jane Grande-Allen^1,*^

^1^Department of Bioengineering, Rice University, Houston, TX

^2^Medical Scientist Training Program, Baylor College of Medicine, Houston, TX

^3^ Department of Surgery, Houston Methodist Hospital, Houston, TX

^4^Center for Cell and Gene, Stem Cells and Regenerative Medicine Center, Translational and Molecular Medicine Program, Baylor College of Medicine, Houston, TX

^5^Morsani College of Medicine, University of South Florida, Tampa, FL

*To whom correspondence should be addressed:

K. Jane Grande-Allen, Department of Bioengineering, Rice University, 6500 Main St., Houston, TX, 77030, [grande@rice.edu](mailto:grande@rice.edu)

Conflicts of Interest: The authors declare no conflicts of interest.

## **Dantrolene has no effect on paVIC f-actin organization**

Since paVICs take on a myofibroblast phenotype during CAVD, we hypothesized that dantrolene may affect cytoskeletal structure via its muscle relaxant properties. We stained cells with phalloidin-488 for 30 min to investigate filamentous actin (f-actin) structure in paVICs treated with LPC, LPC + dantrolene, or vehicle. However, we found no measurable differences in f-actin arrangement due to LPC or dantrolene (**Supplemental Figure 1A-C**).


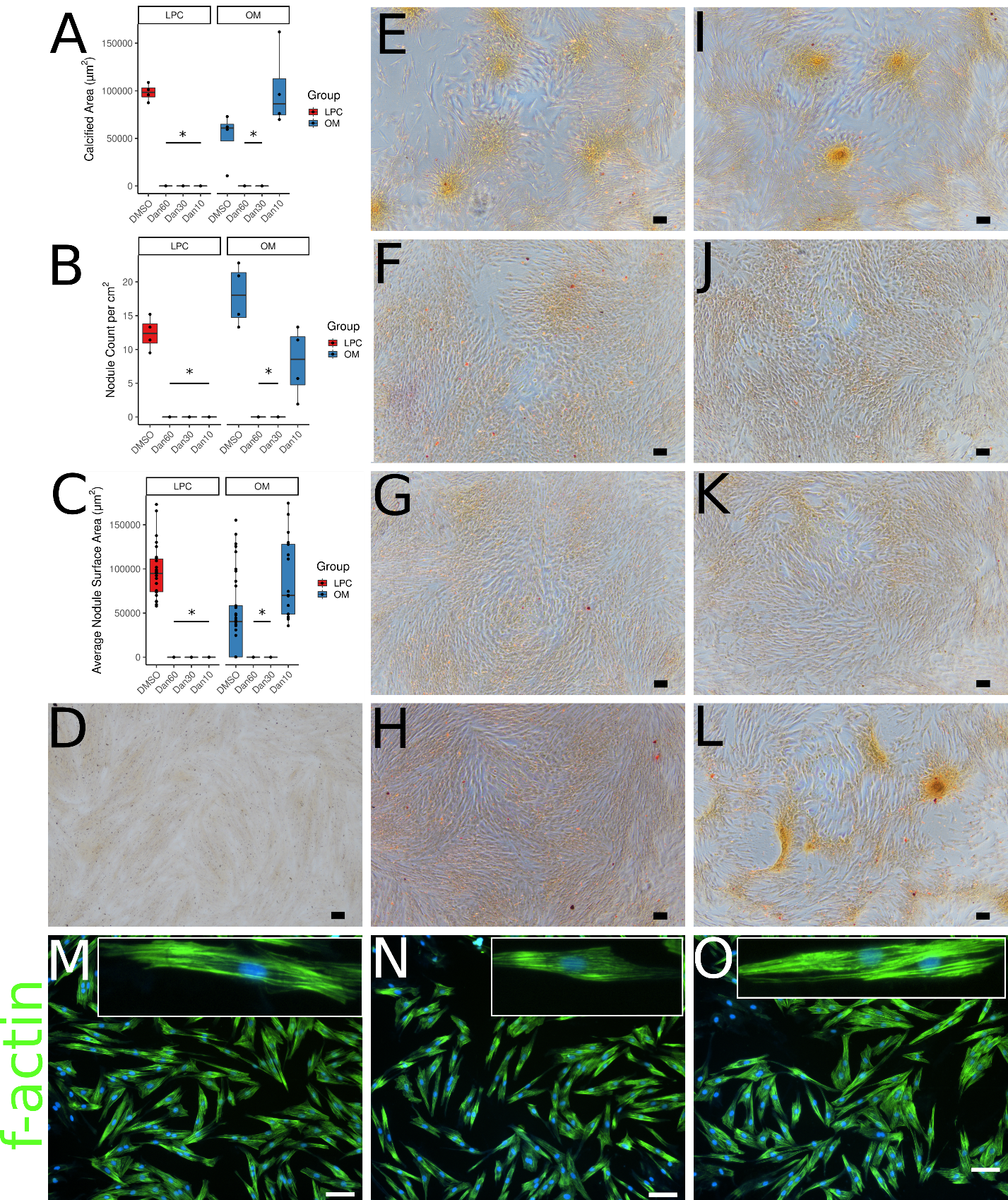


C

A
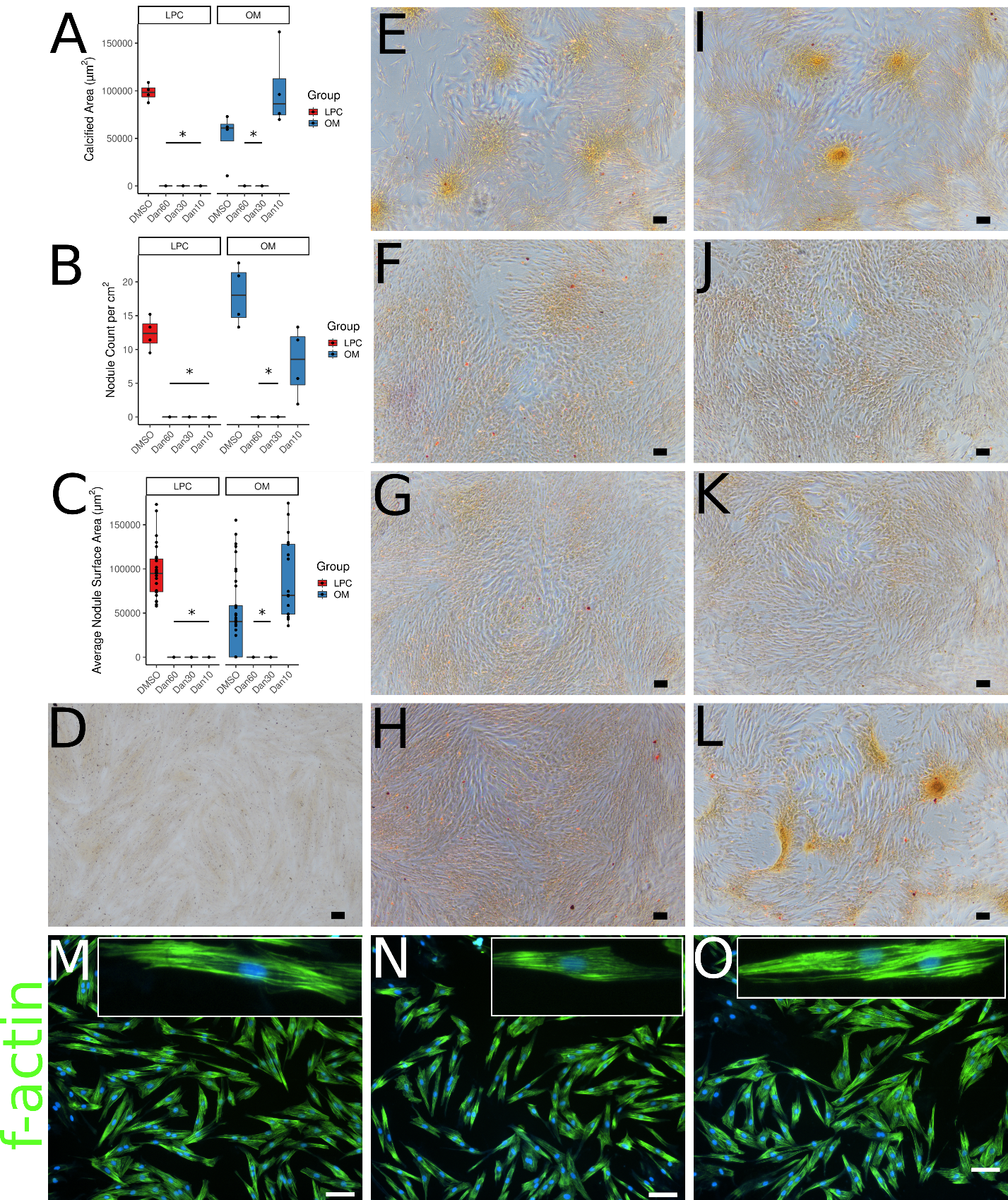


B

**Supplemental Figure 1.** (A) Vehicle control. (B) LPC 10 μM. (C) LPC 10 μM and dantrolene 60 μM. Scale bar shows 100 μm.

## **Dantrolene and LPC have no effect on wound healing**

Others have found that dantrolene-mediated ryanodine inhibition may increase fibroblast-mediated wound healing as measured by a scratch assay.(17) To determine the effects of LPC and dantrolene on paVICs, we performed a scratch assay. Briefly, paVICs were grown to confluence in 6-well plates, then incubated overnight in starving medium. The next day, a central area of the confluent cells was scratched with a micropipette tip. The cells were washed and imaged using an automated microscope stage to image the same areas in each well. The cells were incubated for two days with vehicle controls, dantrolene, and LPC and imaged at 24 and 48 hours However, we found no significant effects of either LPC or Dantrolene, separately or in combination, in wound closure in an *in vitro* scratch assay at 24 hours (**Supplemental Figure 2**). By 48 hours, all samples had fully filled the void left by the scratch.


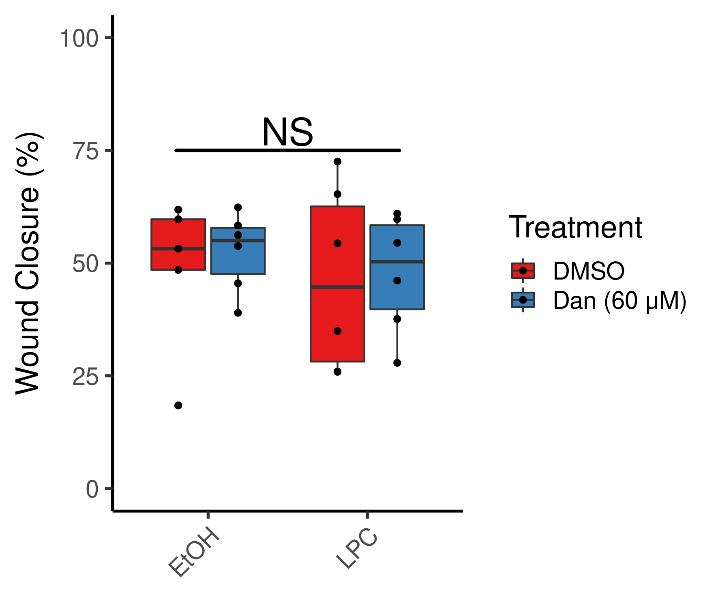


**Supplemental Figure 2.** Dantrolene and LPC do not impact paVIC migration in a scratch assay.

## **Hydrogel Synthesis and Characterization**

To determine if the *in vitro* effects of dantrolene on paVICs transferred to more complex models, paVICs were encapsulated in gelatin methacrylate (Huma OsteoGelMA, Huma Biologics). Briefly, cells were suspended in a monomer solution of 7.5% (w/v) OsteoGelMA in a previously described white light-activated photoinitiator.(15) Then, they were exposed to a brilliant white light for 90s to polymerized the gels. Before cellular experiments optimal compressive modulus and swelling ratio of the hydrogels were determined by creating a series of acellular hydrogels at varying weight fractions. Compressive modulus was measured on an Electroforce 3000 series mechanical tester (Bose). Swelling ratios were determined by weighing the hydrogels immediately after synthesis and overnight after swelling in PBS as previously described.(14) The optimal mechanical conditions were decided based on the parameters described by Puperi et al.(16) After determining the optimal parameters, paVICs were encapsulated in the hydrogels and exposed to media conditions as in other experiments. Gene expression changes were quantified via qRT-PCR. RNA was extracted from the cellular hydrogels after the experimental course by flash freezing them in TRIzol, homogenizing them using a TissueLyser II (Qiagen), and then purifying the supernatant via the methods described above. After making cDNA libraries, qRT-PCR was performed as above. (**Supplemental Figure 3**)


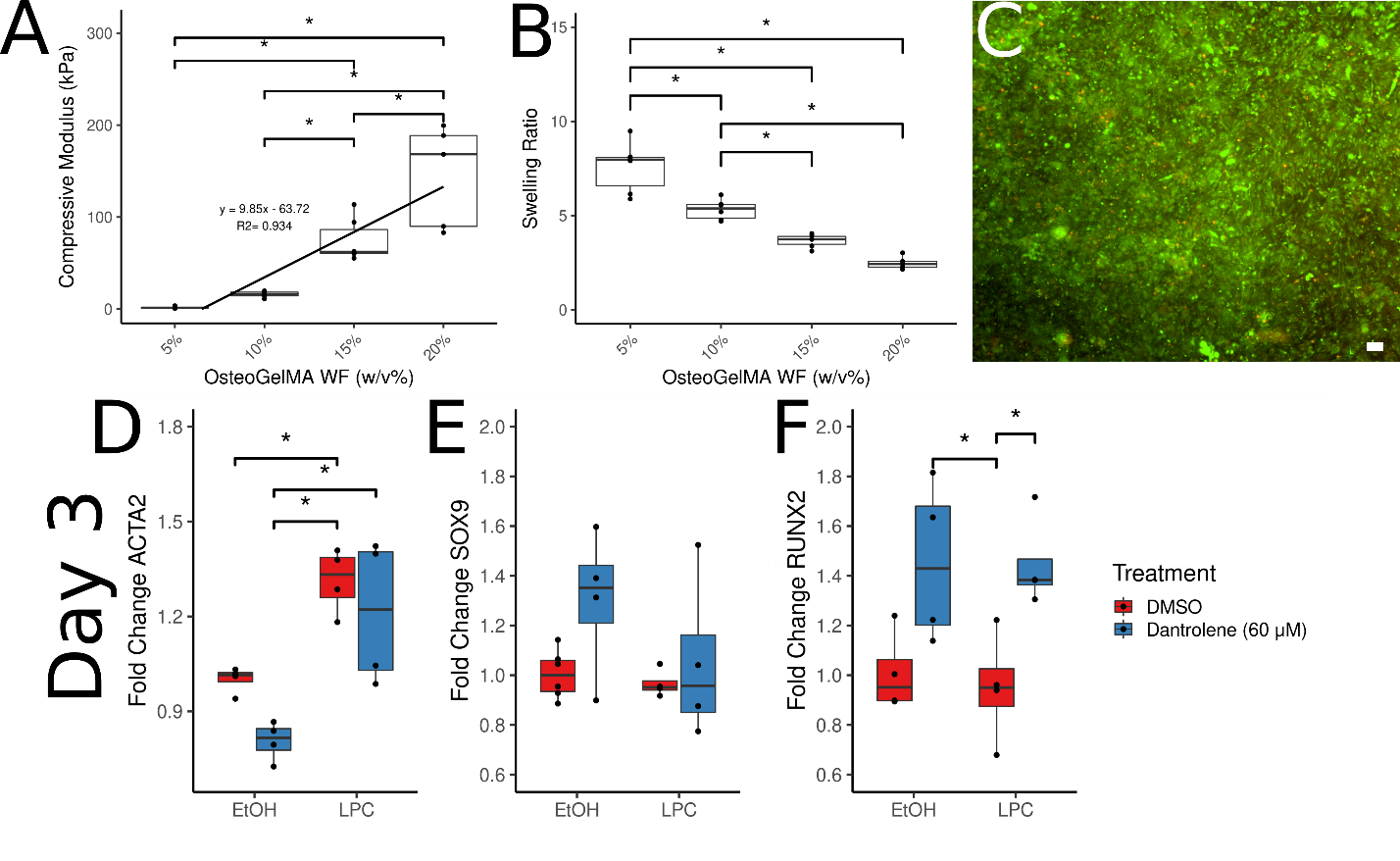
**Supplemental Figure 3. A 3-dimensional microenvironment differentially regulates the effects of LPC and dantrolene on paVICs.** (A) Compressive mechanical testing and (B) swelling ratios were used to determine the optimal weight fraction of OsteoGelMA for paVIC encapsulation. (C) paVICs demonstrate vibrant survival when encapsulated within OsteoGelMA. (D) LPC upregulates *ACTA2* in OsteoGelMA-encapsulated paVICs, an effect which is not reversed by dantrolene. (E) LPC has no effet on *SOX9* in encapsulated paVICs. (F) Dantrolene persistently upregulates *RUNX2* in paVICs, even when encapsulated. (* indicates p < 0.05 via Tukey’s test between the indicated groups; green = living cell, orange = dead cell, scale bar is 100 μm)

**Supplementary Table S1:** Probes used for qPCR.

| Probe | Manufacturer | Target |
| --- | --- | --- |
| Ss04245588_m1 | Applied Biosciences | *ACTA2* |
| qSscCID0002170 | Bio-Rad | *RUNX2* |
| Ss03392406_m1 | Applied Biosciences | *SOX9* |
| Ss03374854_g1 | Applied Biosciences | *GAPDH* |
